# A Teleost Bactericidal Permeability-Increasing Protein Kills Gram-Negative Bacteria, Modulates Innate Immune Response, and Enhances Resistance against Bacterial and Viral Infection

**DOI:** 10.1371/journal.pone.0154045

**Published:** 2016-04-22

**Authors:** Yuan-yuan Sun, Li Sun

**Affiliations:** 1 Key Laboratory of Experimental Marine Biology, Institute of Oceanology, Chinese Academy of Sciences, Qingdao, China; 2 Laboratory for Marine Biology and Biotechnology, Qingdao National Laboratory for Marine Science and Technology, Qingdao, China; 3 University of Chinese Academy of Sciences, Beijing, China; University of Pittsburgh, UNITED STATES

## Abstract

Bactericidal/permeability-increasing protein (BPI) is an important factor of innate immunity that in mammals is known to take part in the clearance of invading Gram-negative bacteria. In teleost, the function of BPI is unknown. In the present work, we studied the function of tongue sole (*Cynoglossus semilaevis*) BPI, CsBPI. We found that CsBPI was produced extracellularly by peripheral blood leukocytes (PBL). Recombinant CsBPI (rCsBPI) was able to bind to a number of Gram-negative bacteria but not Gram-positive bacteria. Binding to bacteria led to bacterial death through membrane permeabilization and structural destruction, and the bound bacteria were more readily taken up by PBL. *In vivo*, rCsBPI augmented the expression of a wide arrange of genes involved in antibacterial and antiviral immunity. Furthermore, rCsBPI enhanced the resistance of tongue sole against bacterial as well as viral infection. These results indicate for the first time that a teleost BPI possesses immunoregulatory effect and plays a significant role in antibacterial and antiviral defense.

## Introduction

The innate immunity system is the first line of host defense against various invaders such as viruses, bacteria, fungi and parasites [1]. It recognizes invading pathogens by the highly conserved pathogen-associated molecular patterns (PAMPs) expressed commonly on microbial organisms. Pattern recognition receptors (PRRs) of the host innate system mediate PAMPs recognition and initiate inflammatory processes [1–3]. As a typical PAMP, lipopolysaccharide (LPS) is one of the bacterial products that most effectively activate innate immune response [4]. It is a major constituent of the outer membrane of Gram-negative bacteria and consists of polysaccharides and a lipid structure named lipid A. Many innate immune-related genes regulated by LPS have been investigated [2]; among them, bactericidal/permeability-increasing protein (BPI) and lipopolysaccharide binding protein (LBP) play crucial roles in the response to the infection of Gram-negative bacteria [5].

BPI and LBP are two structurally and functionally related glycoproteins and were originally isolated from rabbit granulocytes and sera, respectively [6,7]. The structure and functions of these proteins have been well studied in mammals. They share two common domains: an N-terminal BPI/LBP/Cholesteryl ester transfer protein (CETP) domain BPI1 and a C-terminal BPI/LBP/CETP domain BPI2 [8–10]. In general, BPI1 binds the lipid A component of LPS, while BPI2 serves to transport LPS [8]. BPI exerts a strong antibacterial activity against Gram-negative bacteria by its high affinity for lipid A; following binding to lipid A, BPI penetrates into the bacterial inner membrane, where it causes a loss of membrane integrity, dissipation of electrochemical gradients, and eventually bacterial death [11,12]. In addition, BPI can opsonize bacteria to enhance phagocytosis by neutrophils [13].

Most work on BPI/LBP has been performed in mammalian systems. In teleost, the function of BPI/LBP is unknown. Inagawa et al. cloned the first fish *LBP/BPI* homolog from rainbow trout *Oncorhynchus mykiss* in 2002 [14]; subsequently, *LBP/BPI*-related genes have been identified in Atlantic cod *Gadus morhua* [15], channel catfish *Ictalurus punctatus* [16], ayu *Plecoglossus altivelis altivelis* [17], and olive flounder *Paralichthys olivaceus* [18]. However, the biological functions of these proteins have not been characterized. In this study, we reported for the first time the *in vitro* and *in vivo* antibacterial properties of a teleost BPI-like protein identified in tongue sole *Cynoglossus semilaevis*, which is one of the most popular and economically important fish species farmed in China.

## Materials and Methods

### Ethics statement

All experiments involving live animals conducted in this study were approved by the Ethics Committee of Institute of Oceanology, Chinese Academy of Sciences.

### Fish

Tongue sole (average 13.6 g) were purchased in March 25, 2015 from Jin Sha Tan Aquatic Products Development Co., Ltd (Qingdao, Shandong Province), located at Xue Jia Dao, Huangdao district of Qingdao city. The fish were clinically healthy and appeared normal. The fish were acclimatized in the laboratory for two weeks and verified to be absent of bacterial and viral pathogens in liver, kidney, and spleen by plate count [19]. During the acclimatization period, the fish were reared under conditions as reported previously [20]. Briefly, the fish were maintained at 20°C in 325-liter tanks containing aerated seawater with pH of 7.9, oxygen >6 mg/L, and ammonia < 0.1mg/L. The density of the fish was maintained at less than 100 fish/tank. Fish were fed daily with commercial dry pellets (purchased from Shandong Sheng-suo Fish Feed Research Center, Shandong, China) at the amount of ~1.2% body weight. Fish were euthanized with tricaine methanesulfonate (Sigma, St. Louis, USA) before tissue collection.

### Sequence analysis

The cDNA sequence (accession no. XP_008316264) and amino acid sequences of CsBPI were analyzed using the BLAST program at the National Center for Biotechnology Information (NCBI) as reported previously [21]. Domain search was performed with the conserved domain search program of NCBI. The theoretical molecular mass and theoretical isoelectric point were predicted by using EditSeq in the DNASTAR (Madison, WI) software package as reported previously [21]. Multiple sequence alignment was created with DNAMAN.

### Construction of pEtCsBPI

pEtCsBPI, which expresses His-tagged recombinant CsBPI (rCsBPI), was created in the same way as reported previously [22]. Briefly, the coding sequence without signal peptide of CsBPI was amplified by PCR with primers F1 (5’–GATATCATGGTAGAAAACCCCGGGATTGAAA– 3’, underlined sequence, EcoRV site) and R1 (5’–GATATCGAAAAACTCATGTGTGTGTTTGGGT–3’, underlined sequence, EcoRV site); the PCR products were ligated with the T−A cloning vector T-Simple (TransGen Biotech, Beijing, China), and the recombinant plasmid was digested with EcoRV to retrieve the *CsBPI*-containing fragment, which was inserted into pET259 [23] at the SwaI site.

### Purification of recombinant proteins, antibody preparation, and immunoblot

Recombinant CsBPI (rCsBPI) and Trx (rTrx) were purified, reconstituted, removed of endotoxin, and concentrated as described previously [24]. rTrx, a His-tagged protein like rCsBPI, was used in this study as a control protein for rCsBPI, because it could be easily purified in the same way as rCsBPI. The concentration of the purified proteins was determined using the Bradford method with bovine serum albumin as a standard. The purified proteins were analyzed by sodium dodecyl sulfate-polyacrylamide gel electrophoresis (SDS-PAGE) and visualized after staining with Coomassie brilliant blue R-250. Mouse antibody against rCsBPI was prepared as reported previously [24]. For immunoblot, peripheral blood leukocytes (PBL) were prepared and cultured with Leibovitz's L-15 medium (Gibco, Carlsbad, USA) containing 10% calf serum (Gibco, Carlsbad, USA) as reported previously [25]; the cells were distributed into a 2 ml centrifuge tube (~10^7^ cells/tube) containing L-15 medium. *Pseudomonas fluorescens* was added to the tube (~10^7^ CFU/ tube). After incubation at 22°C for 2 h. the extracellular and whole-cell proteins were prepared and used for Western blot as reported previously [25].

### Binding of rCsBPI to bacterial cells

Binding of rCsBPI to the Gram-negative bacteria (*Edwardsiella tarda*, *P*. *fluorescens*, *Vibrio anguillarum*, and *Vibrio harveyi*) and the Gram-positive bacteria (*Micrococcus luteus*, *Staphylococcus aureus*, and *Streptococcus iniae*) was determined by ELISA and immunofluorescence microscopy as reported previously [24].

### Effect of rCsBPI on the survival and membrane/structure integrity of target bacteria

Bactericidal activity of rCsBPI was performed as reported previously [26]. Briefly, *E*. *tarda*, *P*. *fluorescens*, *S*. *aureus*, and *S*. *iniae* were cultured in Luria-Bertani broth (LB) media and resuspended to 1 × 10^4^ CFU/ml in LB medium. 100 μl of the cell suspension was mixed with 200 μg/ml rCsBPI, rTrx, or PBS. The cells were incubated at 22°C for 1 h, 2 h, or 4 h, and bacterial survival was examined by plate count. Membrane permeability was determined by propidium iodide (PI) uptake assay as reported previously [27]. To examine structural change of bacterial cells, P. fluorescens was resuspended in PBS to 5×10^8^ CFU/ml; the cells were incubated with or without (control) rCsBPI or rTrx (200 μg/ml) for 2 h and 4 h at 22°C. The cells were observed with a scanning electron microscope (S-3400N, Hitachi, Japan). All experiments were performed three times.

### Phagocytosis

Phagocytosis determined by fluorescence activated cell sorting (FACS) was performed as reported previously [28]. Briefly, 1 ml fluorescein isothiocyanate (FITC)-labeled *P*. *fluorescens* (10^8^ CFU) was mixed with or without 20 μg rCsBPI or rTrx and incubated at 22°C for 1 h. The cells were washed and resuspended in L-15 medium to 1 × 10^9^ CFU/ml. One milliliter PBL (~10^7^ cells) was mixed with 100 μl untreated *P*. *fluorescens*, rCsBPI-treated *P*. *fluorescens*, rTrx-treated *P*. *fluorescens*, or L-15 medium (control). The mixtures were incubated in the dark for 2 h, and the cells were collected by centrifugation and washed with PBS for three times. Extracellular fluorescence was quenched, and the cells were analyzed with a Partec CyFlow Counter (Partec GmbH, Munster, Germany) as reported previously [28]. The experiment was repeated three times.

### Quantitative real time reverse transcription-PCR (qRT-PCR)

To determine *CsBPI* expression by qRT-PCR under normal conditions, intestine, muscle, brain, blood, heart, liver, kidney, gill, and spleen were taken aseptically from tongue sole and used for total RNA extraction with EZNA Total RNA Kit (Omega Bio-tek, Doraville, GA, USA) as reported previously [21]. qRT-PCR was carried out in an Eppendorf Mastercycler (Eppendorf, Hamburg, Germany) using the SYBR ExScript qRT-PCR Kit (Takara, Dalian, China) [21]. The expression level of *CsBPI* was analyzed using comparative threshold cycle method (2^−ΔΔCT^) with beta-actin (ACTB) as an internal reference [21]. The entire experiment (i.e. extraction of RNA, cDNA synthesis, and qRT-PCR) was performed three times, each time with five fish.

*CsBPI* expression during bacterial and viral infection was determined as reported previously [24]. Briefly, *P*. *fluorescens* was cultured in LB medium at 28°C to mid-logarithmic phase and resuspended in PBS to 1 × 10^7^ CFU/ml. Two groups (20/group) of tongue sole were injected intraperitoneally (i.p.) with 50 μl *P*. *fluorescens* or PBS. At 6 h, 12 h, 24 h, and 48 h post-infection (hpi), *CsBPI* expression in the kidney and spleen of the fish was determined by qRT-PCR as above. For viral infection, megalocytivirus RBIV-C1 [29] was suspended in PBS to 5 × 10^5^ copies/ml; tongue sole were injected i.p. with 50 μl megalocytivirus or PBS as above. At 1 d, 3 d, 5 d and 7 d post-infection (dpi), five fish were taken for tissues collection, and *CsBPI* expression was determined by qRT-PCR as above.

To determine the expression of immune genes interleukin (IL) -1β, IL-6, IL-8, tissue necrosis factor (TNF) α, the chemokines CsCCK1 and CsCXCe1 [30,31], Myd88, CsISG15 [32], high mobility group protein CsHMG [21], and three IRFs (IRF7, 8, and 9), three groups (5/group) of tongue sole were injected intraperitoneally with 50 μl PBS or with rCsBPI or rTrx (200 μg/ml), and the expression of immune genes in head kidney was determined at 12 h post-injection by qRT-PCR as above. All experiments were performed three times.

### *In vivo* infection in the presence of rCsBPI

*In vivo* infection was carried out as reported previously [24]. Briefly, tongue sole were infected with *P*. *fluorescens* or megalocytivirus alone, or with *P*. *fluorescens* or megalocytivirus in the presence of 200 μg/ml rCsBPI or rTrx. Kidney and spleen were taken from the fish (five at each time point) at 12 h, 24 h, and 48 h post-bacterial infection and at 1 d, 3 d, and 5 d post-viral infection. Bacterial recovery from the tissues was determined by plate count, and viral numbers in the tissues was determined by absolute quantitative real time PCR [29]. The experiment was performed three times.

### Statistical analysis

All experiments were performed at least three times, and statistical analyses were carried out with SPSS 17.0 software (SPSS Inc., Chicago, IL, USA). Data were analyzed with analysis of variance (ANOVA), and statistical significance was defined as *P* < 0.05.

## Results

### Characteristics of CsBPI sequence

**Fig 1:**
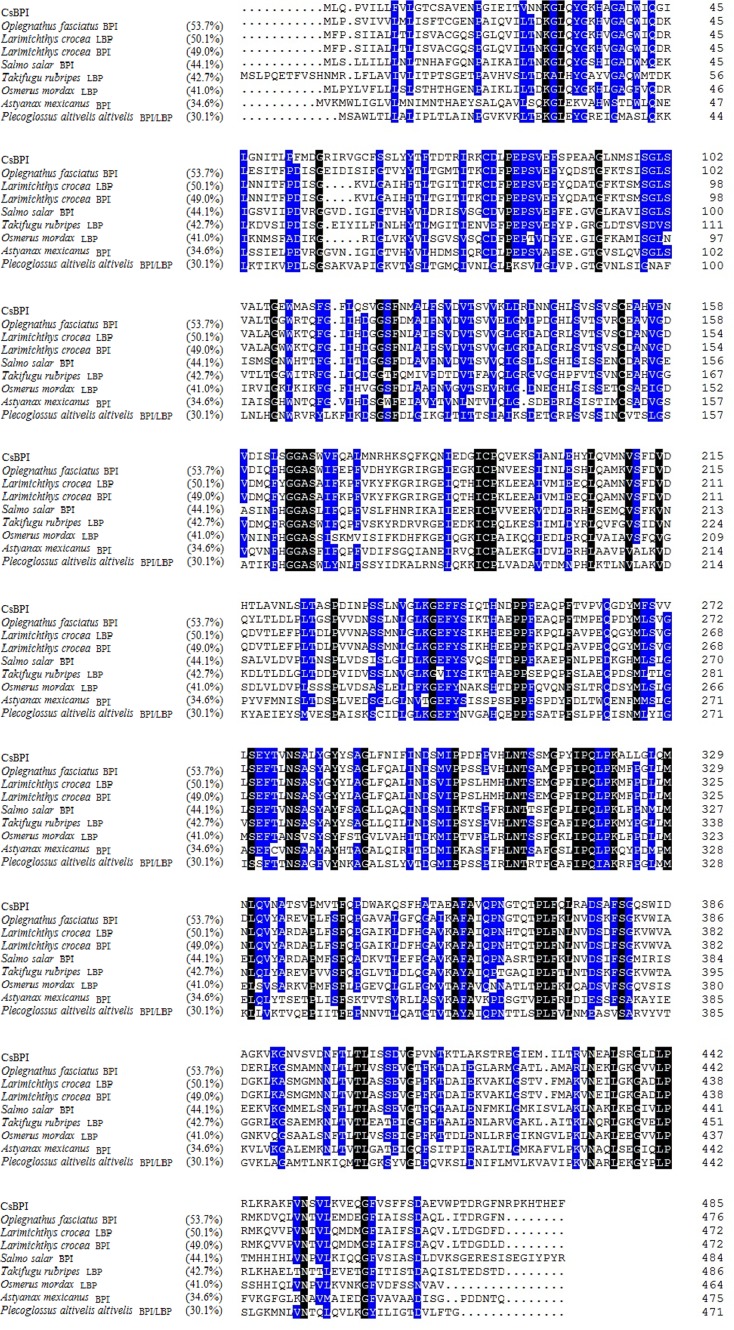
Alignment of the sequences of CsBPI homologues. Dots denote gaps introduced for maximum matching. Numbers in brackets indicate overall sequence identities between CsBPI and the compared sequences. The consensus residues are in black; the residues that are ≥75% identical among the aligned sequences are in blue. The GenBank accession numbers of the aligned sequences are as follows: *Oplegnathus fasciatus*, BAM21037.1; *Larimichthys crocea*, KKF08800.1; *Larimichthys crocea*, ABO32254.1; *Salmo salar*, NP_001135199.1; *Takifugu rubripes*, XP_003976395.2; *Osmerus mordax*, ACO08980.1; *Astyanax mexicanus*, XP_007237045.1; *Plecoglossus altivelis altivelis*, BAG49475.1.

The deduced amino acid sequence of CsBPI contains 486 residues and has a theoretical molecular mass of 53.6 kDa and a pI of 5.8. CsBPI possesses an N-terminal signal peptide (residues 1 to 16), an N-terminal BPI/LBP/CETP domain BPI1 (residues 26–248), and a C-terminal BPI/LBP/CETP domain BPI2 (residues 263–465). CsBPI shares 30.1% to 53.7% overall sequence identities with the known BPI/LBP of a number teleost fish including *Oplegnathus fasciatus*, *Larimichthys crocea*, *Salmo salar*, *Takifugu rubripes*, *Osmerus mordax*, *Astyanax mexicanus*, *Plecoglossus altivelis altiveli* (Fig 1).

### Expression of *CsBPI* in fish tissues in the absence and presence of microbial infection

**Fig 2:**
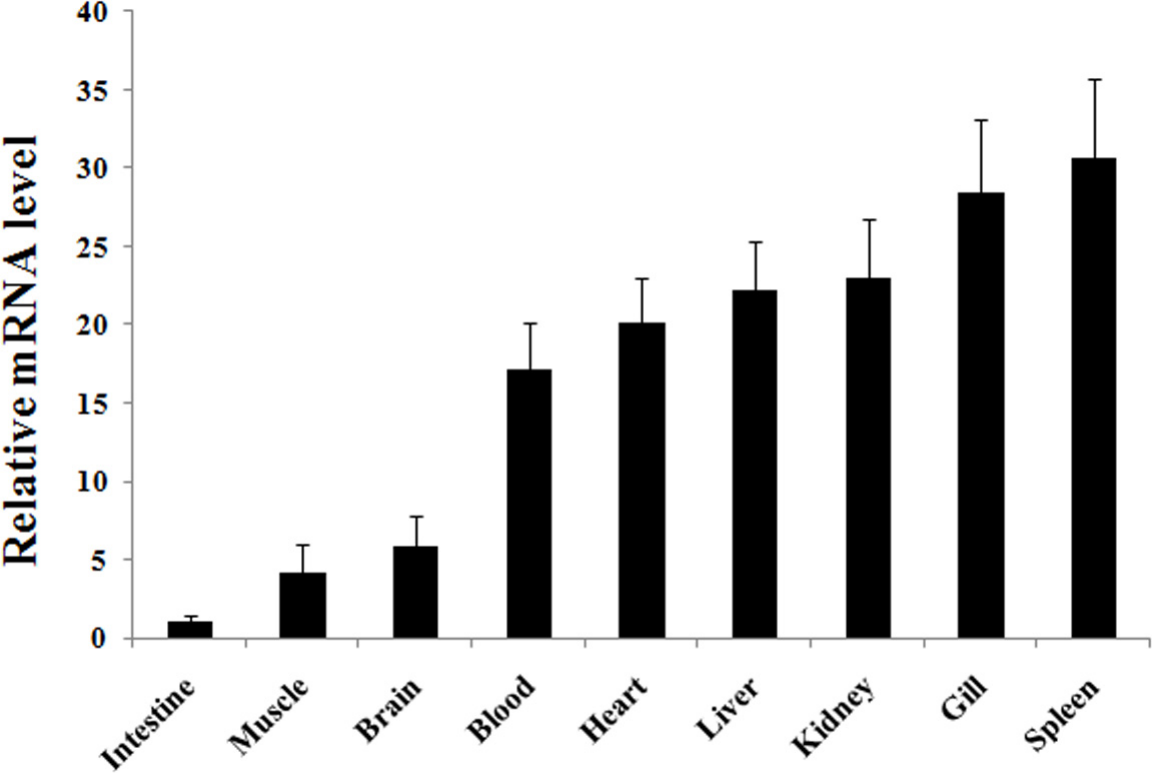
Tissue specific expression of *CsBPI*. *CsBPI* expression in the intestine, muscle, brain, blood, heart, liver, kidney, gill, and spleen of tongue sole was determined by quantitative real time RT-PCR. For convenience of comparison, the expression level in intestine was set as 1. Vertical bars represent means ± SEM (N = 3). N, the number of times the experiment was performed.

**Fig 3:**
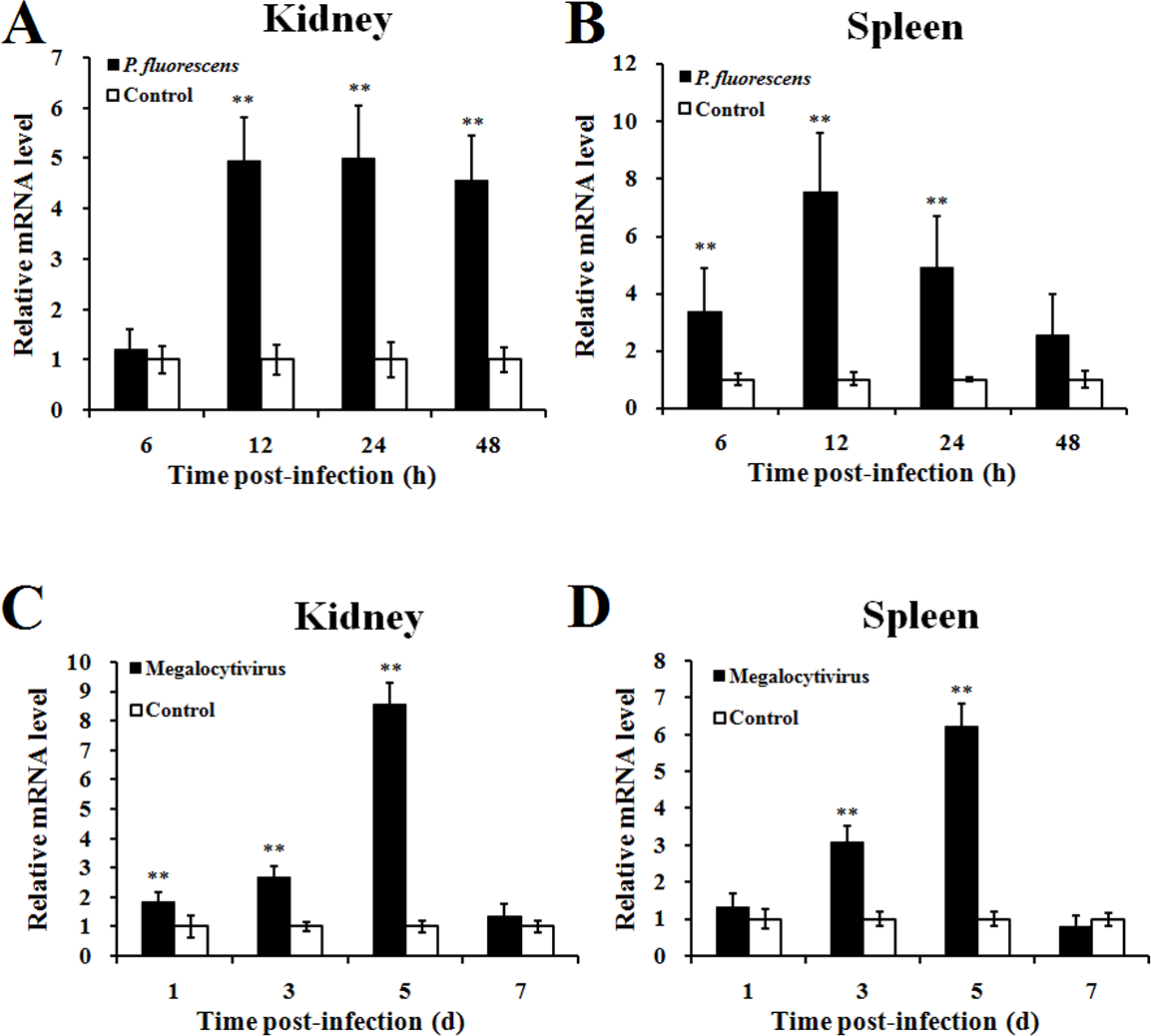
*CsBPI* expression in response to bacterial and viral infection. Tongue sole were infected with *Pseudomonas fluorescens* (A and B), megalocytivirus (C and D), or PBS (control), and *CsBPI* expression in kidney (A and C) and spleen (B and D) was determined by quantitative real time RT-PCR at various time points. For convenience of comparison, the expression level of the control fish was set as 1. Values are shown as means ± SEM (N = 3). N, the number of times the experiment was performed. **P < 0.01.

qRT-PCR analysis revealed that, in the absence of pathogen challenge, *CsBPI* expression occurred, in increasing order, in the intestine, muscle, brain, blood, heart, liver, kidney, gill, and spleen of tongue sole, with the expression level in spleen was 30.7-fold of that in intestine (Fig 2). In the presence of bacterial infection induced by experimental challenge with *P*. *fluorescens*, *CsBPI* expression was significantly upregulated at 12 hpi, 24 hpi, and 48 hpi in kidney, and at 6 hpi, 12 hpi, and 24 hpi in spleen (Fig 3). In the presence of viral infection induced by experimental challenge with megalocytivirus, *CsBPI* expression was significantly upregulated at 1 dpi, 3 dpi, and 5 dpi in kidney, and at 3 dpi and 5 dpi in spleen (Fig 3).

### Preparation of rCsBPI antibody and detection of CsBPI production in PBL

To prepare rCsBPI, the coding sequence of CsBPI was sub-cloned into and expressed in *E*. *coli*, and rCsBPI was purified from the *E*. *coli* host as a His-tagged protein (S1 Fig). Mouse antibody against rCsBPI was then prepared and used to examine CsBPI production in PBL. The results showed that CsBPI was detected in both the extracellular milieu and the whole-cell fraction (S2 Fig).

### Interaction of rCsBPI with Gram-negative and Gram-positive bacteria

**Fig 4:**
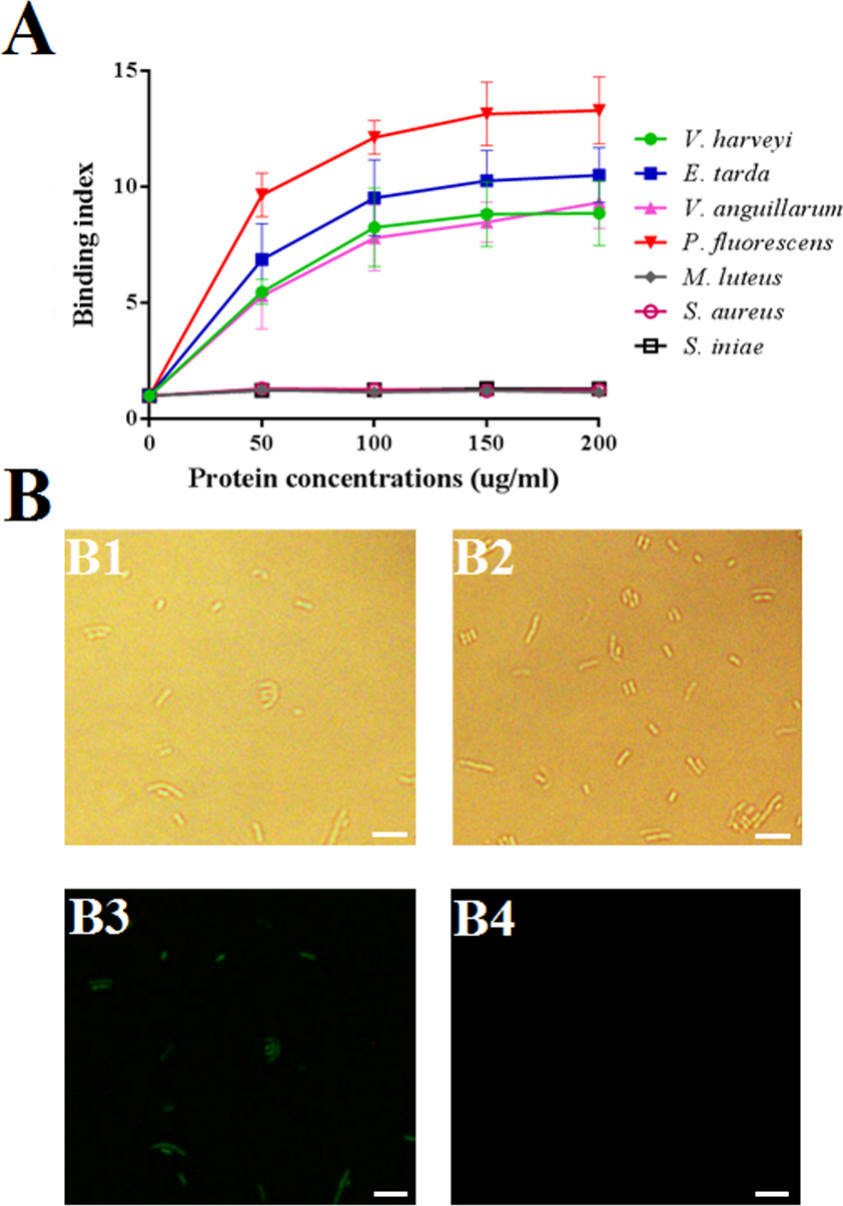
**Binding of rCsBPI to bacteria detected by ELISA (A) and microscopy (B).** (A) *Edwardsiella tarda*, *Pseudomonas* *fluorescens*, *Vibrio anguillarum*, *Vibrio harveyi*, *Streptococcus iniae*, *Micrococcus luteus*, and *Staphylococcus aureus* were incubated with or without (control) different concentrations of rCsBPI, and binding of the protein to bacterial cells was determined by ELISA. Values are shown as means ± SEM (N = 3). N, the number of times the experiment was performed. (B) *P*. *fluorescens* was incubated with rCsBPI (B1 and B3) or rTrx (B2 and B4), and the cells were treated with anti-His antibody and FITC-labeled secondary antibody. The cells were then examined under a fluorescence microscope with (B3 and B4) or without (B1 and B2) fluorescence light. Bar: 4 μm.

When incubated with various Gram-negative (*E*. *tarda*, *P*. *fluorescens*, *V*. *anguillarum*, and *V*. *harveyi*) and Gram-positive (*M*. *luteus*, *S*. *aureus*, and *S*. *iniae*) bacteria, rCsBPI was found to bind to all Gram-negative bacteria in a dose-dependent manner, with the highest binding index being observed with *P*. *fluorescens* (Fig 4A). In contrast, no apparent binding was observed with Gram-positive bacteria. Fluorescence microscopy showed that rCsBPI, but not rTrx, which was purified under the same condition as rCsBPI, bound *P*. *fluorescens* (Fig 4B).

### Bactericidal activity of rCsBPI

**Fig 5:**
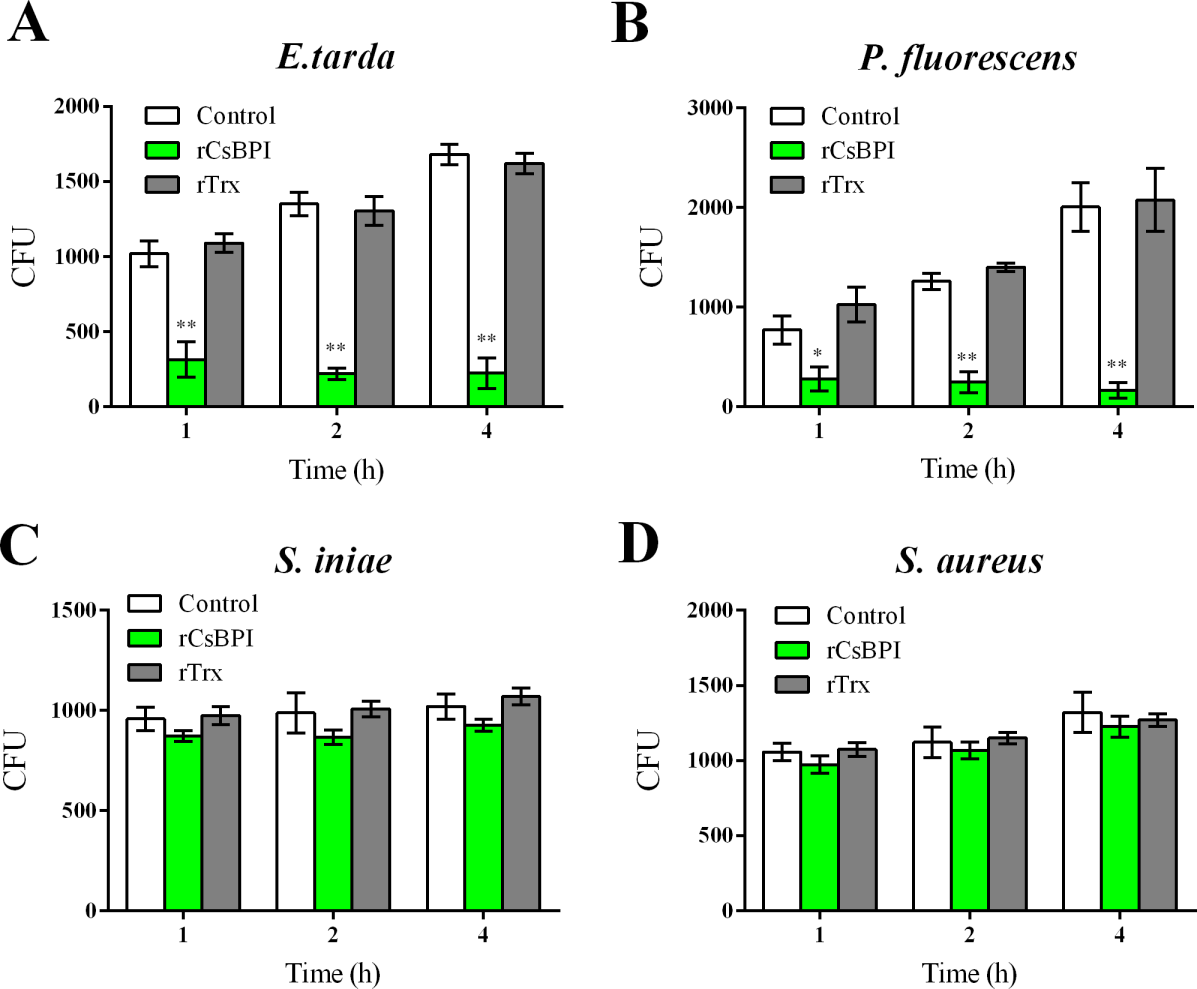
Bactericidal activity of rCsBPI. *Edwardsiella tarda*, *Pseudomonas fluorescence*, *Streptococcus iniae*, and Staphylococcus aureus were incubated with or without (control) rCsBPI or rTrx for different hours. After incubation, the number of survived bacterial cells (shown as Colony Forming Unit, CFU) was determined by plate count. Values are shown as means ± SEM (N = 3). N, the number of times the experiment was performed. **P < 0.01, *P < 0.05.

To examine whether rCsBPI possessed bactericidal activity and whether this activity required direct bacterial interaction, two rCsBPI-interactive bacteria (*E*. *tarda* and *P*. *fluorescence*) and two rCsBPI-non-interactive bacteria (*S*. *iniae* and *S*. *aureus*) were treated with rCsBPI for 1 h, 2 h, or 4 h, and bacterial survival was determined by plate count. The results showed that for both *E*. *tarda* and *P*. *fluorescence*, treatment with rCsBPI, but not with rTrx, dramatically reduced the amount of viable cells at all examined time points (Fig 5A and 5B). In contrast, treatment of *S*. *iniae* and *S*. *aureus* with rCsBPI had no significant effect on bacterial survival (Fig 5C and 5D).

### rCsBPI-induced alteration in the integrity of bacterial membrane and structure

**Fig 6:**
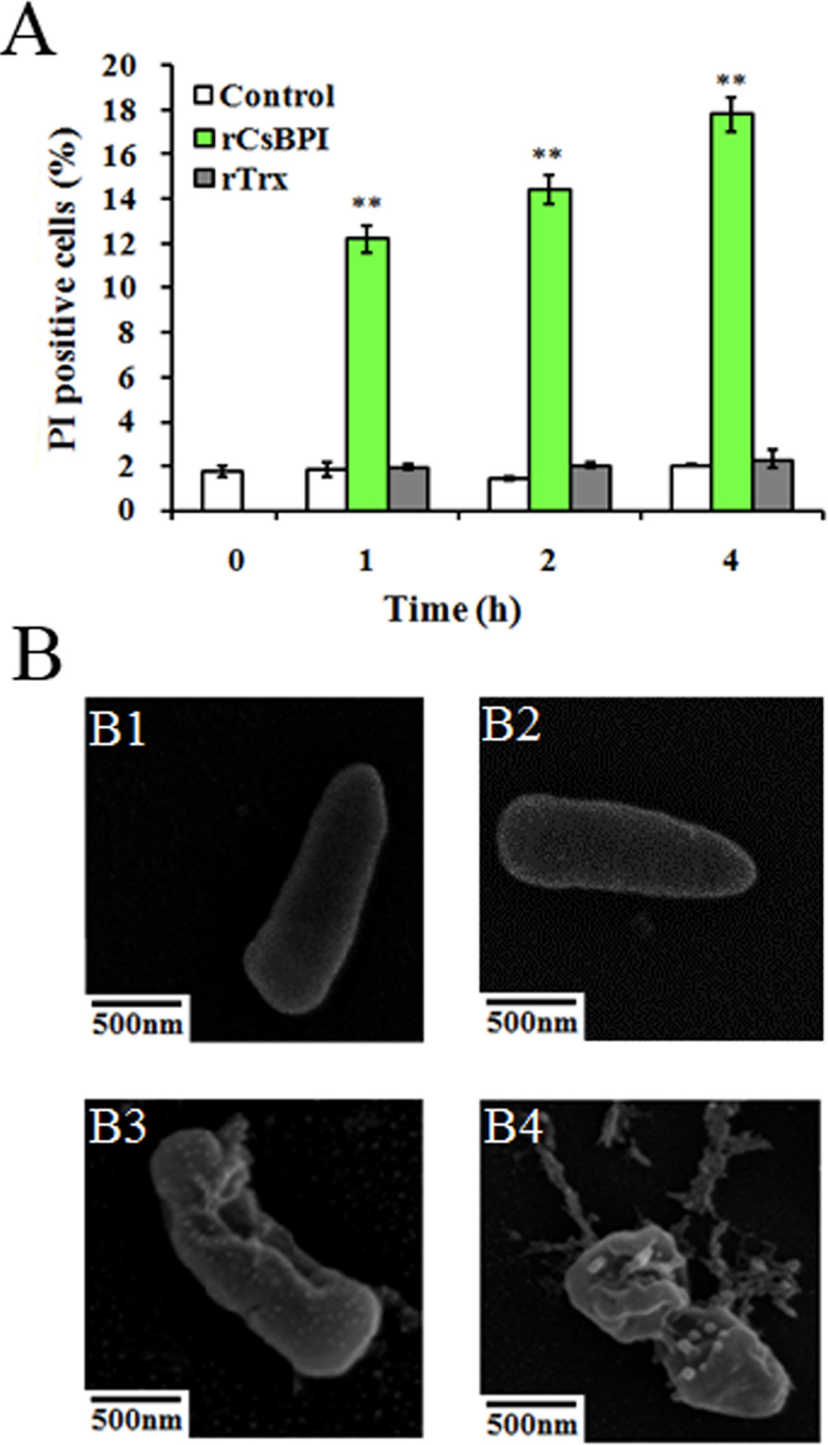
**Effect of rCsBPI on bacterial membrane permeability (A) and cellular structure (B).** (A) *Pseudomonas* *fluorescens* was incubated with or without (control) rCsBPI or rTrx for different hours. After incubation, the cells were stained with PI solution, and PI-positive cells were determined. Values are shown as means ± SEM (N = 3). N, the number of times the experiment was performed. (B) P. fluorescens was treated with rCsBPI for 2 h (B3) and 4 h (B4) or treated with rTrx for 4 h (B2); the control cells (B1) were untreated. The cells were then subjected to microscopy. Bar, 500 nm. **P < 0.01.

To investigate whether the above observed bacterial killing was the result of damaging the structure of the target bacteria by rCsBPI, *P*. *fluorescens* was examined for membrane permeability via PI staining. The results showed that compared to untreated *P*. *fluorescens*, rCsBPI-treated bacteria exhibited a significantly higher amount of PI-positive cells, which increased with the time of treatment (Fig 6A). Microscopic examination revealed that 2 h of rCsBPI treatment induced apparent structural alteration in *P*. *fluorescens*, which became more severe as the time of treatment increased from 2 h to 4 h (Fig 6B). In contrast, rTrx-treated *P*. *fluorescens* were similar to the control cells in both the number of PI-positive cells and cellular morphology.

### Phagocytosis of rCsBPI-bound bacteria

**Fig 7:**
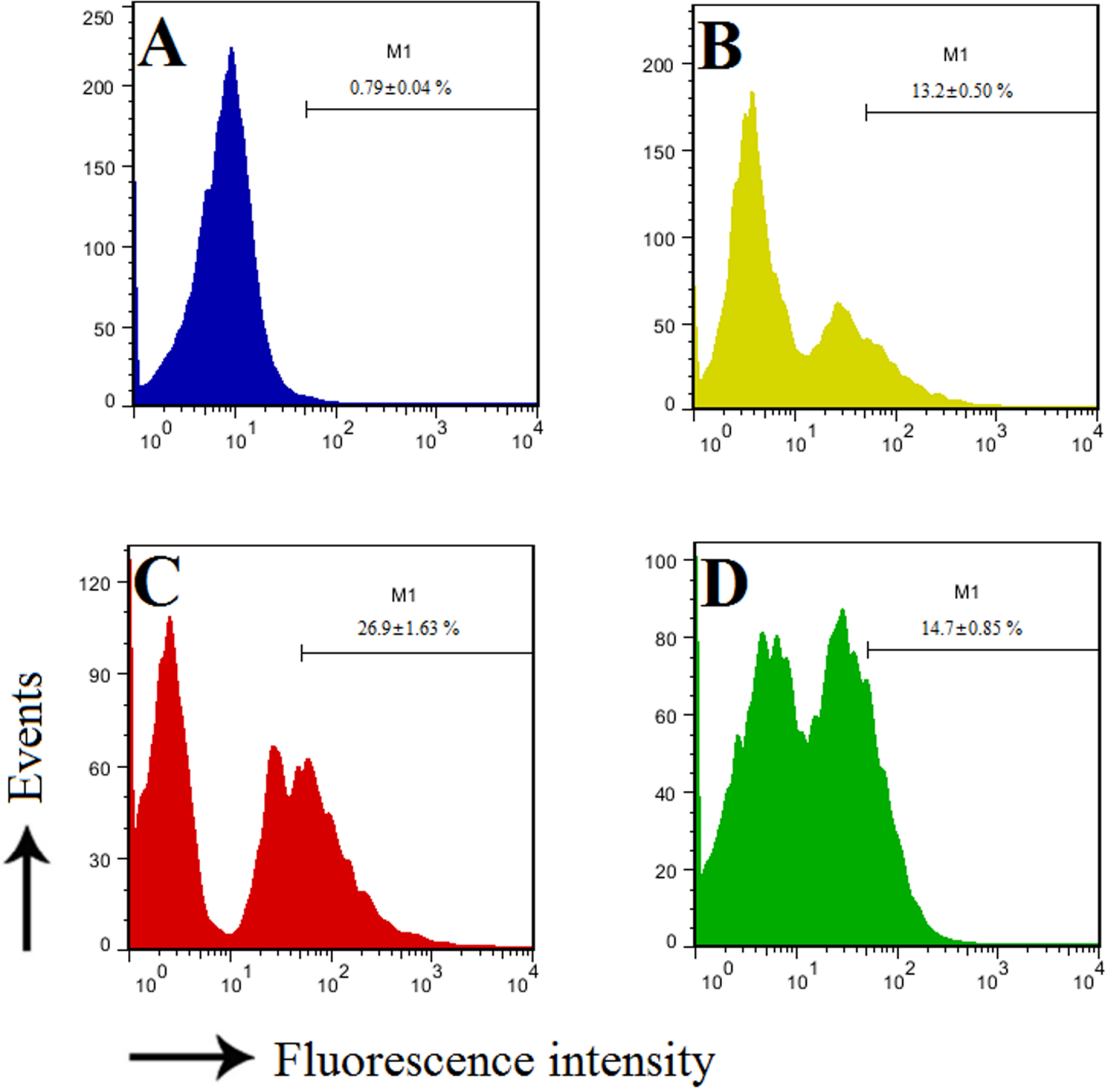
Effect of rCsBPI on phagocytosis. Tongue sole peripheral blood lymphocytes were incubated with FITC-labeled *Pseudomonas* *fluorescens* in the absence (B) or presence of rCsBPI (C) or rTrx (D); the control cells (A) were incubated without bacteria. The cells were then analyzed by fluorescence activated cell sorting. M1 represents the cellular population with enhanced fluorescence as a result of bacterial uptake. Values are shown as means ± SEM (N = 3). N, the number of times the experiment was performed.

To examine whether binding of rCsBPI to bacteria influenced phagocytosis of the bacteria by host immune cells, *P*. *fluorescens* treated with or without rCsBPI were incubated with tongue sole PBL, and bacterial uptake by PBL was subsequently analyzed with FACS. The results revealed that phagocytosis of rCsBPI-treated bacteria (M1 = 26.9% ± 1.63%) was significantly (*P* < 0.01) higher than that of phagocytosis of untreated bacteria (M1 = 13.2% ± 0.50%). Treatment of the bacteria with rTrx had no apparent effect on phagocytosis (M1 = 14.7% ± 0.85%) (Fig 7).

### *In vivo* effect of rCsBPI on immune gene expression and bacterial and viral infection

**Fig 8:**
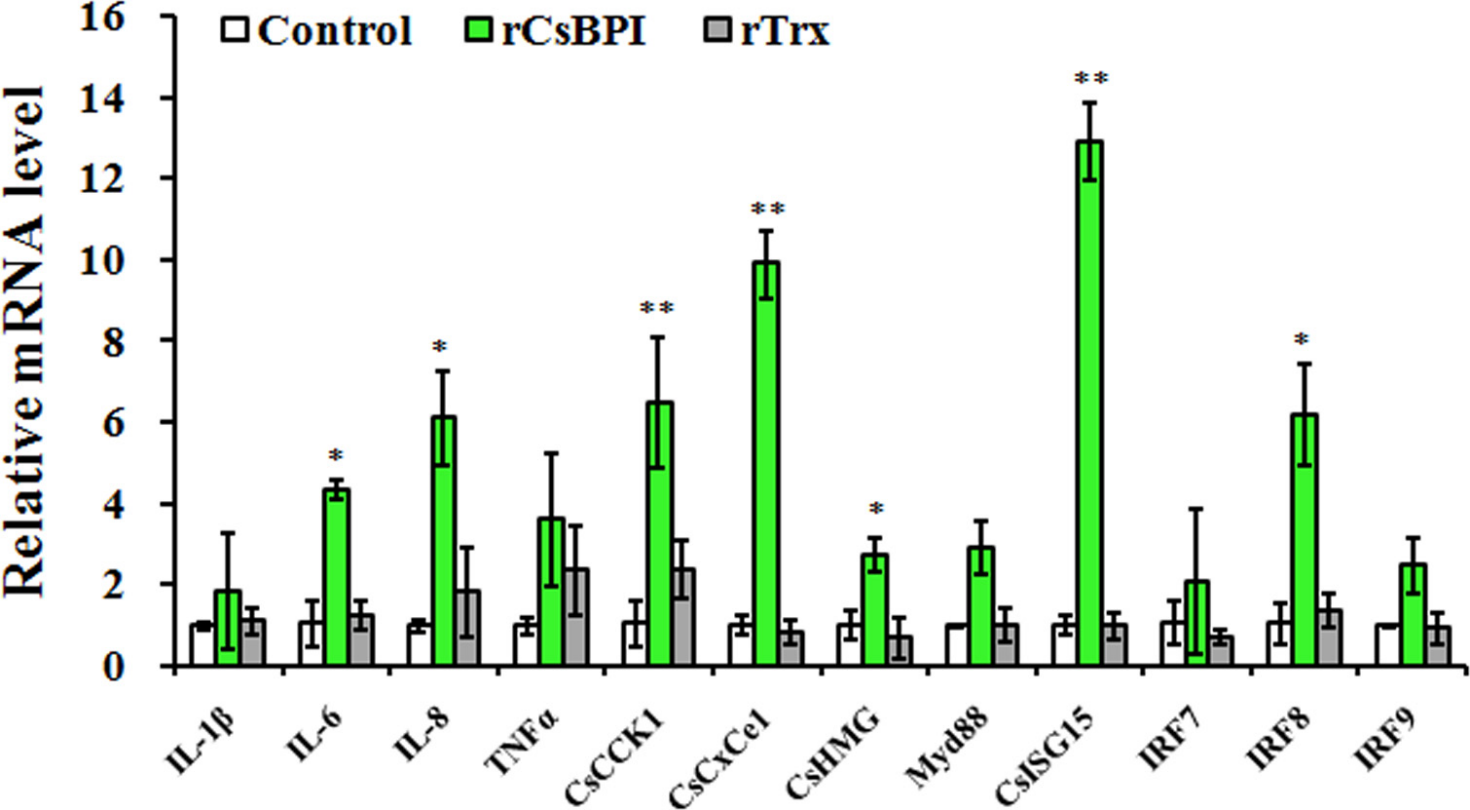
Effect of rCsBPI on the expression of immune genes in tongue sole. Tongue sole were administered with rCsBPI, rTrx, or PBS (control), and expression of the immune genes in head kidney was determined by quantitative real time RT-PCR. In each case, the expression level of the control fish was set as 1. Values are shown as means ± SEM (N = 3). N, the number of times the experiment was performed. **P < 0.01, *P < 0.05.

**Fig 9:**
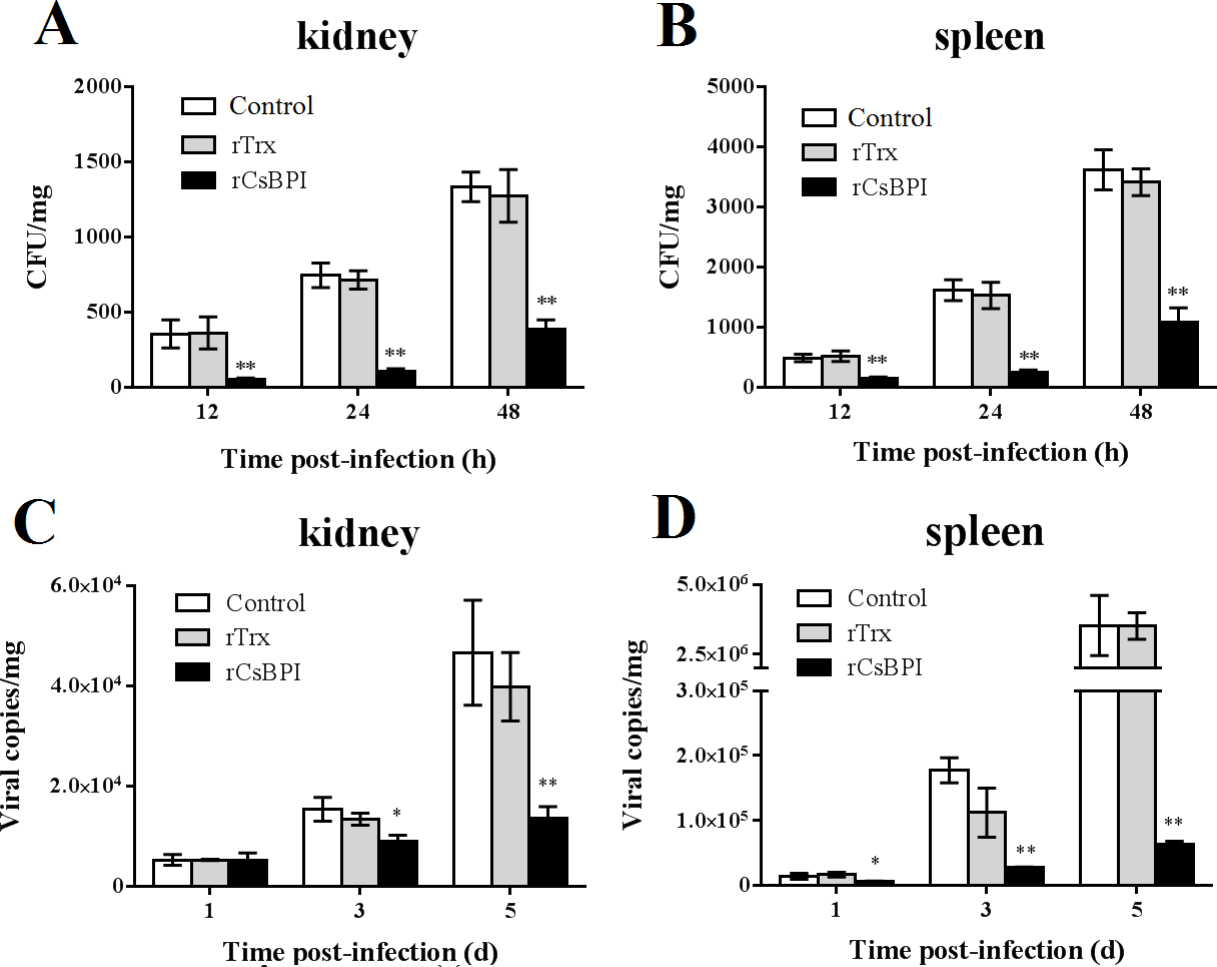
Effect of rCsBPI on bacterial and viral infection in tongue sole. Tongue sole were infected with Pseudomonas fluorescens (A and B) or megalocytivirus (C and D) in the presence or absence (control) of rCsBPI or rTrx. At various time points post-infection, pathogen loads in the kidney and spleen of the fish were determined. Values are shown as means ± SEM (N = 3). N, the number of times the experiment was performed. **P < 0.01, *P < 0.05.

With the above *in vitro* observations, we wanted to investigate the *in vivo* effect of rCsBPI. Previous studies showed that some antimicrobial proteins possess immunoregulatory effects and can modulate immune gene expression [27,33]. In this study, we examined the potential effect of rCsBPI on the expression of immune genes, i.e. IL-1β, IL-6, IL-8, TNFα, two chemonkines (CsCCK1 and CsCXCe1), Myd88, ISG15, the high mobility group protein CsHMG, and three IRFs (IRF7, 8, and 9). The results showed that following administration of rCsBPI into tongue sole, the expression levels of IL-6, IL-8, CsCCK1, CsCXCe1, CsHMG, CsISG15, and IRF8 were significantly increased in the head kidney of the fish (Fig 8). We next examined the effect of rCsBPI on pathogen infection. For this purpose, tongue sole were inoculated with *P*. *fluorescens* or megalocytivirus in the presence of rCsBPI, and pathogen invasion into kidney and spleen were subsequently assessed. The results indicated that in fish infected with *P*. *fluorescens* plus rCsBPI for 12 h, 24 h, and 48 h, the bacterial loads in kidney and spleen were significantly reduced compared to the control fish (Fig 9A and 9B). Similarly, in fish infected with megalocytivirus plus rCsBPI, the viral burdens in both tissues were significantly decreased at 3 d pi and 5 d pi compared to control fish (Fig 9C and 9D). In contrast, rTrx had no apparent effect on either immune gene expression or pathogen infection.

## Discussion

In this study, we analyzed the sequence structure, expression profile, and biological function of a teleost BPI-like protein, CsBPI. Mammalian *BPI* is known to be expressed in neutrophils and various mucosal epithelia [34,35]. In teleost, expression of *BPI/LBP* has been detected in a wide arrange of tissues such as gill, intestine, kidney, spleen, and blood [16,17,36]. In our study, qRT-PCR analysis showed that under normal physiological conditions, *CsBPI* was expressed in multiple tissues, in particular immune organs. Similar to previous reports that *BPI/LBP* expression is enhanced by stimulation with microbial pathogens [15,37], we found that challenge with *P*. *fluorescens* and megalocytivirus induced significant upregulation of *CsBPI* expression in fish tissues. These results indicate that CsBPI is involved in the immune response elicited by both bacterial and viral pathogens.

In mammals, BPI is an extracellular protein secreted by cells such as polymorphonuclear leukocytes (PMN) and acts as a primary antibacterial effector [38]. In our study, we found that CsBPI exhibits a putative signal peptide sequence and was predicted to be an extracellular protein. Consistently, after *P*. *fluorescens* infection of PBL, CsBPI was detected in the culture medium of the cells, suggesting that like mammalian BPI, CsBPI is produced into the extracellular milieu by PBL upon pathogen stimulation.

As a member of the classical PRRs, BPI is known to participate in antimicrobial defense toward Gram-negative bacteria by damaging the inner and outer bacterial membranes [7,11,39]. The N-terminal domain of BPI contains functional regions proposed to be involved in LPS binding and bactericidal activity [40–42]. In fish, the antibacterial potential of BPI has not been investigated. In this study, we found that rCsBPI exhibited different binding capacities to Gram-negative bacteria including *P*. *fluorescens*, *V*. *anguillarum*, *E*. *tarda*, and *V*. *harveyi*, all which are serious pathogens to a wide range of fish species. In agreement with these results, bactericidal activity of rCsBPI was detected only with Gram-negative bacteria. The highest binding index and bactericidal activity of rCsBPI were observed with P. fluorescens, which suggests the possibility that the LPS structure of P. fluorescens may differ from the other tested bacteria in aspects such as accessibility to rCsBPI. Similar to the BPI of humans and oyster [12,43], which display a permeabilizing effect on bacterial membranes, rCsBPI induced damage of the membrane of *P*. *fluorescens* and time-dependent destruction of cellular structures.

Previous reports showed that BPI can serve as an opsonin for the phagocytosis of Gram-negative bacteria by neutrophils [44,45]. In a model of *Streptococcus pneumoniae* lung infection, recombinant human BPI21 was shown to improve the survival of challenged mice by enhancing the apoptosis of upper respiratory tract cells and macrophage–bacteria interaction [46]. Another report demonstrated that human BPI played a role in the delivery of bacteria to phagocytic cells, and only bacteria preincubated with BPI were ingested by neutrophils and monocytes [47]. In contrast, the immune function of fish BPI/LBP, in particular that under *in vivo* condition, is unknown. In our study, we observed that treatment of *P*. *fluorescens* with rCsBPI significantly increased the uptake of the bacteria by PBL, suggesting that rCsBPI facilitated the process of phagocytosis. Consistently, *in vivo* study showed that fish treated with rCsBPI exhibited significantly lower bacterial loads in tissues following *P*. *fluorescens* infection, suggesting that rCsBPI reduced bacterial dissemination into and colonization of host tissues. The anti-infection effect of rCsBPI is likely due to the following reasons: (i) during the process of co-administration of rCsBPI and *P*. *fluorescens* in the infection experiment, some bacteria may be directly killed by rCsBPI before having a chance of tissue invasion; (ii) some bacteria may be bound by rCsBPI and thus easily uptaken and cleared by phagocytes. In addition, we detected in rCsBPI-treated fish enhanced expression of a number of immune genes involved in proinflammatory response (IL-6 and IL-8), antibacterial immunity (CsCCK1 and CsCXCe1) [30,31], antiviral immunity (CsISG15) [32], and both antibacterial and antiviral immune respones (CsHMG and IRF8) [19,21]; the upregulated expression of all these genes very likely contributes to the ability of rCsBPI to inhibit bacterial as well as viral infection. Considering that rCsBPI, being a protein, may not have a very long half-life *in vivo* due to degradation by host proteases, it is possible that the anti-infection effect of rCsBPI observed at the later infection stages (such as that after 24 h) is mediated by the antibacterial/antiviral immune response induced by rCsBPI.

In both mammals and fish, antiviral activity of BPI/LBP has not been documented. In the current study, we found that tongue sole infected with megalocytivirus in the presence of rCsBPI exhibited significantly reduced viral burdens in tissues, suggesting that rCsBPI exerted an inhibitory effect on viral replication. This is the first indication of an antiviral potential associated with BPI. In the absence of any evidence of direct interaction between rCsBPI and the virus, it is likely that the inhibitory effect of rCsBPI on viral infection is the result of rCsBPI-induced expression of the genes, such as IRF8, involved in antiviral immunity.

In conclusion, in this study we demonstrated that CsBPI is a secreted immune factor with bacteria-binding and bactericidal activities against Gram-negative bacteria. Like mammalian BPI, rCsBPI increases the membrane permeability of target bacteria and promotes phagocytosis by host immune cells. However, unlike known BPI, rCsBPI exhibits novel properties unreported previously, i.e. immunoregulatory effect and the ability to inhibit not only bacterial but also viral infection *in vivo*, though the effect on virus is likely an indirect one mediated by rCsBPI-induced antiviral immune response. These results add new insights into the biological function of BPI.

## Supporting Information

S1 Fig SDS-PAGE analysis of purified recombinant proteins. Purified rCsBPI and rTrx (lanes 1 and 2, respectively) were analyzed by SDS-PAGE and viewed after staining with Coomassie brilliant blue R-250. M, protein markers.
(TIF)

S2 Fig Production of CsBPI in peripheral blood leukocytes (PBL). Proteins prepared from the extracellular and whole-cell (lanes 2 and 3 respectively) factions of tongue sole PBL were analyzed by immunoblot with antibody against rCsBPI (A), rTrx (B), or β-actin (C). Lane 1: protein markers.
(TIF)
