# Supplementary figures and images for: A Teleost Bactericidal Permeability-Increasing Protein Kills Gram-Negative Bacteria, Modulates Innate Immune Response, and Enhances Resistance against Bacterial and Viral Infection
Source: PLoS One. 2016 Apr 22;11(4):e0154045. doi: 10.1371/journal.pone.0154045 (PMC4841584; doi:10.1371/journal.pone.0154045)

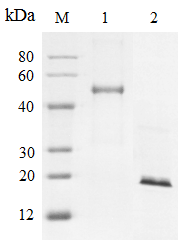

Supplement: S1 Fig — Purified rCsBPI and rTrx (lanes 1 and 2, respectively) were analyzed by SDS-PAGE and viewed after staining with Coomassie brilliant blue R-250. M, protein markers. (TIF) [file pone.0154045.s001.tif]

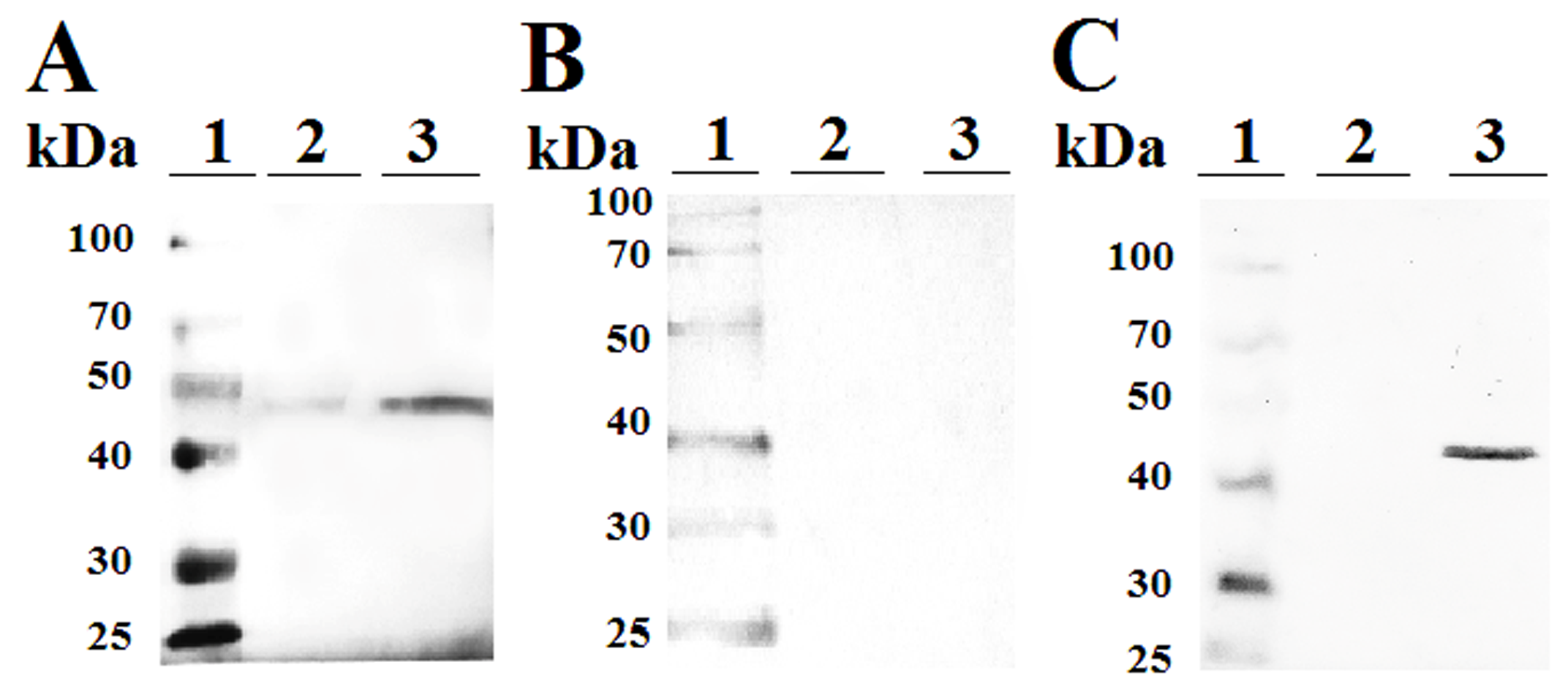

Supplement: S2 Fig — Proteins prepared from the extracellular and whole-cell (lanes 2 and 3 respectively) factions of tongue sole PBL were analyzed by immunoblot with antibody against rCsBPI (A), rTrx (B), or β-actin (C). Lane 1: protein markers. (TIF) [file pone.0154045.s002.tif]
